# Defending is a Balancing Act: The Consequences of Defending Victims of Bullying Depend on Defender’s Popularity and Social Preference

**DOI:** 10.1007/s10964-026-02363-4

**Published:** 2026-05-30

**Authors:** Daniek H. J. Joosten, Rozemarijn van der Ploeg, Antonius H. N. Cillessen, Bram O. de Castro

**Affiliations:** 1 Research Institute of Child Development and Education, University of Amsterdam, Amsterdam, The Netherlands; 2 Department of Pedagogy and Educational Sciences, University of Groningen, Groningen, the Netherlands; 3 Behavioural Science Institute, Radboud University Nijmegen, Nijmegen, The Netherlands

**Keywords:** Defending, Victimization, Social Status, Peer Norms, Classroom Norms

## Abstract

Defending peers who are victimized by bullying is often promoted as a prosocial strategy, yet the conditions under which defending protects defenders versus exposes them to social risks are not well understood, especially in primary school. This study examined when defending predicts increased versus decreased risk for defenders by considering individual status (popularity, social preference) and classroom-level norms as moderators. Participants were 9439 Dutch primary school students (49.1% female; *M*age = 10.55 years, *SD* = 1.47, range = 6.84–15.62) from 433 classrooms in 161 schools. Data were collected at the beginning and end of one school year (September, 2017 – July, 2018), including self-reports of victimization and bullying and peer nominations of defending, victimization, bullying, popularity, and social preference. Multilevel models showed no consistent main effect of defending on self-reported victimization. Instead, the social consequences of defending depended strongly on defenders’ peer status: defending predicted higher victimization for highly popular youth and for youth low in social preference, whereas defending predicted lower victimization for youth low in popularity and for those high in social preference. Classroom bullying descriptive norms were associated with higher overall levels of victimization, but classroom norms related to bullying and defending did not moderate the association between defending and victimization. Sensitivity analyses using peer-reported victimization indicated a small positive association between defending and defenders’ victimization as perceived by classmates. Together, these findings indicate that defending is embedded in peer status hierarchies and classroom climates and that the risks and benefits of defending depend on defenders’ position within the peer group.

**Supplementary Information:**

The online version contains supplementary material available at 10.1007/s10964-026-02363-4.

## Introduction

Bullying in school is typically defined as systematic, intentionally harmful behavior characterized by a power imbalance between bullies and victims (Volk et al., 2014). It is a pervasive problem associated with substantial short- and long-term maladjustment for both victims and perpetrators, including internalizing problems, academic difficulties, and later externalizing and psychiatric problems (Cunningham et al., 2016; Schoeler et al., 2018; Ye et al., 2023). Bullying does not occur in isolation, however; it unfolds within a broader peer ecology in which bystanders play a central role in maintaining or challenging harmful social dynamics (Salmivalli et al., 2010). In this respect, defending victimized peers is often seen as a promising strategy to reduce bullying and improve victims’ adjustment, yet defending may also expose defenders themselves to social risks. However, evidence on when defending is protective versus risky for defenders remains limited and mixed, particularly in primary school and with respect to the joint roles of individual status and classroom norms. This study addresses this gap by examining concurrent and longitudinal associations between defending and victimization in primary school classrooms, and by testing how these associations depend on children’s position in the peer group and the normative climate of their classroom.

Defending is usually defined as comforting or supporting behaviors toward victims during or after bullying episodes, in which contemporary research distinguishes between at least two broad forms of defending: victim-oriented and bully-oriented defending (Meter & Card, 2015; Malamut et al., 2025). Victim-oriented defending includes behaviors such as comforting the victim, offering emotional support, or involving adults on the victim’s behalf, whereas bully-oriented defending involves directly confronting or challenging the perpetrator (Gini et al., 2008). These forms are likely to carry different social dynamics and risks. Publicly confronting a bully may involve more salient challenges to status and a higher risk of retaliation than quietly supporting a victim, even though both forms can signal opposition to bullying.

Importantly, a consistent finding is that defending, independently of which form, benefits victims. Compared with undefended victims, those who have at least one defender report higher self-esteem, stronger feelings of belonging, and higher peer acceptance with less rejection (Laninga-Wijnen et al., 2023; Saarento et al., 2015; Sainio et al., 2011). Moreover, by encouraging prosocial behaviors, defending may also help create healthier social hierarchies in which respect and inclusion are prioritized over coercion and dominance, fostering a more positive and collaborative peer environment (Sainio et al., 2011). In contrast, when peers witness bullying but do not intervene, victims may experience heightened distress, anxiety, and depressive symptoms (Ye et al., 2023), and inaction may reinforce a passive peer environment that normalizes bullying (Pozzoli & Gini, 2013). For these reasons, many school-based interventions encourage defending behavior as a central strategy to address bullying (Salmivalli et al., 2021).

At the same time, however, many youth perceive defending as risky. Specifically, because intervening may prevent bullies from achieving their goals, undermine their control over social resources, or challenge their dominance, and therefore potentially provoke retaliation from them and their allies (Huitsing et al., 2014; Olthof et al., 2011). Moreover, according to the “retaliation hypothesis”, bullies may target defenders to punish those who threaten their position, reinforcing the belief that defending carries social costs rather than rewards.

While youth may be apprehensive to defend others out of fear of retaliation, research findings on *actual* increases in victimization following defending actions are mixed. Defending was concurrently associated with higher rates of victimization (e.g., Lambe et al., 2019), but effect sizes were small and only significant when considering youth self-reports (Ma et al., 2019). Importantly, these concurrent findings cannot be interpreted causally, so it is unclear whether defending actually leads to future victimization or whether children who are already victimized tend to defend. The few longitudinal empirical investigations that have been conducted found opposing results: defending was a risk factor for future victimization in one study (Huitsing et al., 2014), but a protective factor against victimization in another study (Meter & Card, 2015). Malamut et al. (2025) tested these associations more rigorously by considering both between-person effects (i.e., differences between children) and within-person effects (i.e., fluctuations within children over time) and found a negative between-person association between comforting defending and self-reported victimization. However, they also could not draw firm conclusions about the underlying peer dynamics.

### Social Status and Peer Norms

One reason for these inconsistent findings may be that the social consequences of defending depend on defenders’ own position within the peer group and on whether their behavior aligns with classroom norms. Status motives are central in peer ecologies: youth often pursue goals of social status and may use both prosocial and aggressive strategies to reach these goals (Hawley, 1999; Veenstra et al., 2010).

Researchers distinguish between two types of social status: Being highly preferred versus being seen as popular (Cillessen & Marks, 2011). Socially preferred youth tend to be prosocial and supportive, whereas popular youth often combine social skills and leadership with more coercive or aggressive tactics to maintain their position (Cillessen & Bellmore, 2022; Rose et al., 2004). Moreover, a recent meta-analysis reported an average correlation of *r* = .45 between social preference and popularity, indicating substantial overlap but also clear divergence between these two constructs (van den Berg et al., 2020). These differences suggest that defending may have distinct implications for socially preferred versus popular youth. Defending may reinforce the prosocial reputation of socially preferred youth, whereas it may challenge or complicate the dominance-based strategies of highly popular youth, potentially exposing them to status challenges or retaliation.

Classroom norms may also shape the social meaning of defending. Descriptive norms capture the average level of a behavior in the classroom, whereas popularity norms reflect the extent to which a behavior is associated with popularity (Dijkstra & Gest, 2015). Youth tend to behave in ways that align with classroom norms because deviating from those norms can entail social costs, and conformity is often associated with greater acceptance (Laursen & Veenstra, 2021; Sentse et al., 2007). When defending is descriptively common or associated with popularity, defending may be viewed as appropriate and may be less risky. In contrast, when bullying is frequent or closely tied to popularity, defending may deviate from the prevailing norms and therefore be more likely to elicit negative reactions. Malamut et al. (2023) examined these processes among Finnish adolescents aged 12–15 years, testing how popularity and classroom norms moderated associations between defending and victimization. Although they did not find a direct longitudinal effect of defending on victimization, they identified concurrent moderation effects: defending was associated with higher victimization in classrooms with low bullying descriptive norms and high bullying popularity norms, and some moderating effects were more pronounced for peer-reported than for self-reported victimization. These findings suggest that the social consequences of defending are embedded in classroom norms and may differ depending on whether victimization is captured through peer reputation or personal experience.

The present study extends this work in several ways. First, it focuses on primary school youth, a developmental period in which peer hierarchies and classroom norms are becoming salient but may still be less crystallized than in middle adolescence (Cillessen & Mayeux, 2004). During late childhood and early adolescence, popularity and social preference begin to diverge as distinct forms of status (Rose et al., 2004), and classroom norms regarding bullying and defending are still in the process of formation. In particular, high status in late childhood appears to be more closely tied to prosocial behavior and academic competence, whereas in adolescence it is more strongly linked to dominance, romantic appeal, and, in some cases, aggression, meaning that behaviors such as defending may carry different social implications at these ages. These developmental characteristics may, therefore, also influence the extent to which defending may shape the social consequences for status and liking (Cillessen & Mayeux, 2004), and whether classroom norms buffer or amplify the risks of defending (Saarento et al., 2015). Second, the study includes both popularity and social preference as distinct indicators of peer status, allowing a more detailed examination of how different dimensions of status relate to the risks and benefits of defending. Third, the study examines both concurrent and longitudinal associations between defending and victimization in a large sample of primary school classrooms, thereby testing whether defending predicts changes in victimization over the course of one school year and whether these associations depend on individual status and classroom norms.

## The Current Study

Despite growing evidence that defending can have both protective and costly consequences, relatively few studies have examined these processes in primary school using longitudinal, multilevel designs that differentiate between popularity and social preference and that incorporate multiple classroom norms. The current study addressed this gap by examining concurrent and longitudinal associations between defending and victimization among primary school youth, focusing on popularity, social preference, and classroom bullying and defending norms as potential moderators. No consistent main effect of defending on victimization was expected, because the association was anticipated to depend on children’s social position and classroom context. Defending was expected to be associated with increased victimization among youth with low social preference and with decreased victimization among youth with high social preference. With respect to popularity, competing possibilities were considered: popular defenders might be protected from retaliation because of their visibility and influence, but they might also be particularly vulnerable if defending conflicts with dominance-based strategies or threatening the status of other high-status peers. The moderating role of popularity was therefore treated as exploratory, with the expectation that any emergent patterns would clarify how different forms of status shape the social consequences of defending. At the classroom level, it was expected that higher bullying descriptive norms and stronger bullying popularity norms would be associated with higher levels of victimization and greater risks for defenders, whereas higher defending descriptive norms and stronger defending popularity norms would be associated with lower overall victimization and reduced risks for defenders.

## Method

### Participants

The sample consisted of 9439 students (49.1% female; *M*_*age*_ = 10.55 years, *SD* = 1.47, range 6.84–15.62 years) from 433 classrooms (*M*_classroom size_ = 22 students, range 10–31 students) in 161 primary schools, who participated in at least one of the two waves. Of these students, 48.4% were in the waiting-list control condition, and 51.6% were in the intervention condition. Most students (92.6%) were born in the Netherlands. Participants were only included if they did not switch classes during the school year and if their classrooms consisted of at least 10 students at T1, in order to reliably assess classroom-level norms (Garandeau et al., 2022).

Of the 9439 students who participated at Time 1, 6259 (66.3%) also provided self-reported victimization data at Time 2. Attrition analyses compared students with data at both waves (retained) to those with T1-only data (drop-out) on age, defending, popularity, social preference, and self-reported victimization at T1, as well as gender (see Table S1). Retained students were younger than drop-out students (*M* = 10.13 vs. 11.59 years, *d* = 1.11). Groups differed only slightly on defending (*M* = 0.17 vs. 0.16, d = 0.06), popularity (*M* = -0.04 vs. −0.00, *d* = 0.11), and victimization (*M* = 0.77 vs. 0.66, *d* = 0.24), and did not differ on social preference (*d* ≈ 0) or gender composition. These patterns indicate that attrition was mainly related to age and only marginally to the substantive variables. Age was therefore included as a covariate in all models.

### Recruitment and Procedure

Participants were part of a larger longitudinal, nationwide investigation on the effectiveness of antibullying programs in the Netherlands in 2017 and 2018 (for a detailed description of the antibullying programs, see de Castro et al., 2018). Schools were either assigned to an intervention condition (after T1) or a waiting-list control condition (after T2). Data were collected twice within the same school year: once at the beginning of the school year, before the start of the interventions (T1; September – October, 2017), and once at the end of the school year, at intervention termination (T2; June - July, 2018), among students in grades 4 to 6 and their teachers. At both waves, students individually completed online questionnaires (45 min) during regular school hours. Video clips informed students about the goals and design of the study, and they were provided with a definition of bullying. Students were assured that their responses would be kept confidential and that they could opt out of the study at any time.

### Measures

#### Individual-level variables

##### Victimization

*Self-reported*. Students’ experienced victimization was assessed using two widely used items from the validated Olweus Revised Bullying/Victimization Questionnaire (Olweus, 1996; Solberg & Olweus, 2003), focusing on the frequency and duration of victimization. Before answering, students watched a short video explaining the definition of bullying, emphasizing intentionality, repetition, and power imbalance (Olweus, 1996), to ensure students had a shared understanding of the construct. Afterwards, they were asked about the frequency of bullying: “*How often have you been bullied since the start of the school year?”* Students could answer on a 5-point scale (1 = *never*, 2 = *once or twice*, 3 = *two or three times a month*, 4 = *about once a week*, 5 = *multiple times a week*). Students who indicated that they were bullied at least “two or three times a month” (i.e., a response of 3 or higher) were subsequently presented with a follow-up question assessing the duration of their victimization: “*How long have you been bullied?*”, with three response options (1 = *since this school year*, 2 = *also last year*, 3 = *for several years*). The self-reported victimization measure used in the analyses was based on this duration question among students who met the frequency criterion (score ≥ 3). Thus, the resulting self-reported victimization scale ranged from 1 to 3, with higher scores indicating more persistent victimization among youth who were bullied frequently enough to receive the follow-up question. Although the response scale was ordinal, responses were treated as a continuous variable in the analyses, consistent with prior research using similar measures (e.g., Laninga-Wijnen et al., 2023). At T2, peer victimization was again assessed with Olweus’ duration item of victimization.

*Peer-reported*. Peer-reported victimization was assessed by asking students to indicate which classmates were “*bullied by other children*” during the past few months. All students could nominate as many or as few classmates as they wanted. Nominations received were counted for each student and standardized within classrooms to account for differences in class size. Higher scores reflected higher levels of peer-reported victimization.

##### Defending

Defending was assessed by asking students, independent of being victimized or not, “*Some children help others who are being bullied. They can comfort or defend them. Which classmates do this*?” All students could nominate as many or as few classmates as they wanted. Nominations received were counted for each student and standardized within classrooms to account for differences in class size. Higher scores reflected higher levels of defending.

##### Bullying

*Peer-reported.* Peer-reported bullying was measured by asking students, “*Who bullies you?”* Students could nominate as many or as few classmates as they wanted. This item captures dyadic aggressor-victim ties, reflecting which classmates are perceived as bullying the respondent, rather than a generalized reputational judgment of “*who bullies other children.”* Nominations received were counted for each student and standardized within classrooms to account for differences in class size. Higher scores reflected greater involvement in bullying as reported by peers.

*Self-reported.* Self-reported bullying was measured using a widely used item from the Olweus Revised Bullying Questionnaire (Olweus, 1996; Solberg & Olweus, 2003). Students were asked: “*How often have you bullied others since the start of the school year?”* Students could answer on a 5-point scale (1 = *never*, 2 = *once or twice*, 3 = *two or three times a month*, 4 = *about once a week*, 5 = *multiple times a week*). Although the response scale was ordinal, responses were treated as a continuous variable in the analyses, consistent with prior research using similar measures. At T2, bullying was again assessed with Olweus’ bullying questionnaire.

##### Popularity

Popularity was measured by asking students, “*Which students are the most popular in your class?”* and “*Which students are the least popular in your class?”* Students could nominate as many or as few classmates as they wanted. For each student, popularity was determined by subtracting the number of least popular nominations received from the number of most popular nominations received (van den Berg et al., 2020). Afterwards, this score was standardized within classrooms to account for differences in class size. Higher scores reflected higher levels of popularity.

##### Social preference

Social preference was assessed by asking students to nominate classmates they *“liked the most*” and “*liked the least*”. Students could nominate as many or as few classmates as they wanted. For each student, preference was determined by subtracting the number of least liked nominations received from the number of most liked nominations received. Afterwards, this score was standardized within classrooms to account for differences in class size. Higher scores reflected higher levels of social preference.

#### Classroom-level predictor variables

##### Bullying norms

*Bullying descriptive norms* were operationalized as the classroom-level average of peer-nominated bullying and computed by averaging the individual proportion scores of bullying of all students in the classroom. *Bullying popularity norms* were calculated as the within-classroom correlation between peer-nominated bullying and peer-nominated popularity. *Bullying social preference norms* were calculated as the within-classroom correlation between peer-nominated bullying and peer-nominated social preference.

##### Defending norms

*Defending descriptive norms* were operationalized as the classroom-level average of peer-nominated defending and computed by averaging the individual proportion scores of defending by all students in the classroom. *Defending popularity norms* were calculated as the within-classroom correlation between peer-nominated defending and peer-nominated popularity. *Defending social preference norms* were calculated as the within-classroom correlation between peer-nominated defending and peer-nominated social preference.

#### Control variables

The present study controlled for intervention status by labeling students in waitlist control schools as 0 and students in intervention schools as 1. The number of students per classroom, students’ age, and gender (boy = 4808; girl = 4631) were also included as control variables.

### Analyses

A series of multilevel models was conducted to examine the concurrent and longitudinal effects of defending on victimization and to test potential moderators at the individual and classroom levels, using the *lme4* package in R (Bates et al., 2015). All models included a random intercept for classroom to account for the nesting of students within classes. Defending, popularity, and social preference were group-mean centered within classrooms (cluster-mean centering) to separate individual-level from classroom-level effects and to account for contextual influences (Enders & Tofighi, 2007), and the corresponding classroom means were included as level-2 predictors.

Separate concurrent and longitudinal models were specified, with self-reported victimization (duration-based measure; see Measures) at the corresponding time point as the dependent variable. For each model set, individual-level predictors were first included to test the direct association between defending and victimization. Next, within-level interaction terms (Defending x Popularity and Defending x Social Preference) and classroom-level moderators (bullying and defending descriptive and popularity norms) were added, followed by cross-level interactions between defending and the classroom-level norms. For the longitudinal analyses, baseline self-reported victimization was included as a covariate to examine whether defending and its moderators predicted changes in victimization over time.

Initial full models included all control variables, main effects, and theoretically relevant interaction terms. Non-significant control variables were removed in a stepwise fashion to obtain more parsimonious models, guided by AIC and BIC values. For key theoretically motivated interaction terms (in particular, the Intervention x Defending interaction), additional likelihood ratio tests comparing nested models were used to evaluate whether these terms improved model fit.

All models were fit using the *lmer* function in R. Additional sensitivity and robustness analyses, including models with alternative classroom norms, Intervention x Defending interactions, and peer-reported victimization as the outcome, are described in the Results section.

#### Missing data and multiple imputation 

Across the main study variables, item-level missingness ranged from 0% to 26.7%. Missingness on the primary outcome variables was modest at T1 (8.1% for self-reported victimization and 6.5% for peer-reported victimization) and higher at T2 (26.7% and 24.3%, respectively), reflecting students who were no longer present at the second assessment. Missing data were handled using multiple imputation in R. Thirty imputed datasets were created using predictive mean matching, including all predictors and outcomes, as well as auxiliary variables related to missingness, in the imputation model. Multilevel analyses were conducted in each imputed dataset, and parameter estimates were pooled according to Rubin’s rules (Little et al., 2014). This approach reduces bias due to missing data and assumes that data are missing at random conditional on the variables included in the imputation model.

#### Sensitivity analyses 

In addition to the primary models, several sensitivity analyses were planned to examine the robustness of the findings, including models that added additional covariates (e.g., peer-reported victimization, self-reported bullying), models with alternative classroom norms, models including Intervention x Defending interaction terms, and models using peer-reported victimization as the outcome. The results of these analyses are reported in the Sensitivity Analyses subsection of the Results and in the online supplement.

### Transparency and Openness

This study reported how it determined the sample size, all data exclusions (if any), all manipulations, and all measures in the study, and the study follows JARS (Appelbaum et al., 2018). All data, analysis code, and research materials are stored in the University of Amsterdam research data repository. Due to institutional regulations, they are not publicly accessible, but they can be made available by emailing the corresponding author. Data were analyzed using R, version 4.5.1 (R Core Team, 2025).

## Results

### Preliminary Analyses

Descriptive statistics and correlations for the main variables are presented in Table 1. All results for the main analyses, including standardized and unstandardized effects, can be found in Table 2. There were small to moderate positive associations of individual-level defending with popularity and social preference at T1 (*r*’s = 0.11 and 0.35), and T2 (*r*’s = 0.09 and 0.29). There were moderate negative associations of victimization with popularity and social preference at T1 (*r* = − .25 and *r = −* .27) and T2 (*r* = − .27 and *r = −* .26). Victimization was relatively stable from T1 to T2 (*r* = .51).

**Table 1:** Descriptive Statistics and Bivariate Correlations among Study Variables

		Descriptives			Correlations										
		M	SD		1	2	3	4	5	6	7	8	9	10	11
**Individual Level**															
	1. Defending	0.16	0.13		—	-0.05***	0.13***	0.37***	-0.04**	0.12***	0.30***	—	—	—	—
					—	<0.001	<0.001	< 0.001	0.003	<0.001	<0.001	—	—	—	—
	2. Self-reported Victimization T1	1.21	0.37		-0.01	—	-0.22***	-0.25***	0.51***	-0.19***	-0.22***	—	—	—	—
					0.193	—	<0.001	<0.001	<0.001	<0.001	<0.001	—	—	—	—
	3. Popularity T1	-0.27	0.29		0.11***	-0.25***	—	0.37***	-0.21***	0.83***	0.31***	—	—	—	—
					< 0.001	< 0.001	—	< 0.001	< 0.001	< 0.001	< 0.001	—	—	—	—
	4. Social Preference T1	0.18	0.24		0.35***	-0.27***	0.37***	—	0.21***	0.31***	0.73***	—	—	—	—
					< 0.001	< 0.001	< 0.001	—	< 0.001	< 0.001	< 0.001	—	—	—	—
	5. Self-reported Victimization T2	1.22	0.37		-0.01	0.51***	-0.24***	-0.24***	—	-0.23***	-0.26***	—	—	—	—
					0.652	< 0.001	< 0.001	< 0.001	—	<0.001	< 0.001	—	—	—	—
	6. Popularity T2	-0.26	0.31		0.09***	-0.22***	0.81***	0.31***	-0.27***	—	0.36***	—	—	—	—
					< 0.001	< 0.001	< 0.001	< 0.001	< 0.001	—	<0.001	—	—	—	—
	7. Social Preference T2	0.20	0.27		0.29***	-0.22***	0.30***	0.71***	-0.26***	0.36***	—	—	—	—	—
					< 0.001	< 0.001	< 0.001	< 0.001	< 0.001	< 0.001	—	—	—	—	—
**Classroom Level**															
	8. Bullying Descriptive Norms	0.08	0.49		0.13***	0.19***	-0.07***	-0.10***	0.17***	-0.06***	-0.05***	—	—	—	—
					< 0.001	< 0.001	< 0.001	< 0.001	< 0.001	< 0.001	< 0.001	—	—	—	—
	9. Bullying Popularity Norms	0.25	0.33		-0.07***	-0.03**	0.03**	-0.02	-0.04**	0.03*	-0.08***	-0.01	—	—	—
					< 0.001	0.002	0.002	0.112	0.004	0.016	< 0.001	0.662	—	—	—
	10. Defending Descriptive Norms	0.17	0.08		0.58***	0.05***	0.02	0.12***	0.04**	0.01	0.13***	0.22***	-0.12***	—	—
					< 0.001	< 0.001	0.084	< 0.001	0.001	0.333	< 0.001	< 0.001	< 0.001	—	—
	11. Defending Popularity Norms	0.17	0.30		0.10***	0.09***	-0.04***	-0.01	0.08***	-0.02	0.02	0.16***	-0.30***	0.17***	—
					< 0.001	< 0.001	< 0.001	0.589	< 0.001	0.112	0.050	< 0.001	< 0.001	< 0.001	—

Note. Descriptives and correlations among the uncentered individual level variables and classroom level variables are below the diagonal. Correlations among the centered individual level variables are above the diagonal. .* *p* < .05. ** *p* < .01, *** *p <* .001

**Table 2:** Multilevel Model Predicting Self-Reported Victimization with Standardized and Unstandardized Effects

Predictor	Concurrent					Longitudinal				
	β	b	SE	t	*p*	β	b	SE	t	*p*
Intercept	0.00	1.38	0.05	27.62	0.000***	0.01	0.79	0.06	13.35	0.000***
Defending T1	0.04	-0.03	0.11	-0.24	0.814	0.05	0.22	0.13	1.65	0.099
Popularity T1	-0.15	-0.21	0.02	-12.88	0.000***	-0.09	-0.12	0.02	-6.59	0.000***
Social Preference T1	-0.21	-0.35	0.02	-16.45	0.000***	-0.11	-0.19	0.02	-7.80	0.000***
Bullying Descriptive Norms	0.13	0.98	0.12	8.08	0.000***	0.06	0.45	0.13	3.49	0.001***
Bullying Popularity Norms	0.01	0.02	0.02	0.92	0.358	0.00	0.01	0.02	0.51	0.612
Defending Descriptive Norms	0.02	0.08	0.08	0.97	0.331	0.02	0.09	0.09	1.03	0.304
Defending Popularity Norms	0.02	0.02	0.02	1.00	0.319	0.00	0.01	0.02	0.43	0.664
Defending T1 * Popularity T1	0.03	-0.22	0.08	2.73	0.006**	0.03	0.41	0.16	-2.57	0.010*
Defending T1 * Social Preference T1	0.00	0.01	0.17	0.08	0.937	-0.04	-0.64	0.20	-3.21	0.001**
Defending T1 * Bullying Descriptive Norms	0.00	0.00	0.71	0.01	0.996	0.00	-0.26	0.82	-0.32	0.749
Defending T1 * Bullying Popularity Norms	0.02	0.16	0.12	1.38	0.169	0.00	0.07	0.13	0.54	0.586
Defending T1 * Defending Descriptive Norms	0.01	0.47	0.47	1.00	0.320	0.00	-0.35	0.56	-0.62	0.536
Defending T1 * Defending Popularity Norms	0.01	0.16	0.12	1.28	0.200	0.00	-0.08	0.14	0.57	0.568

Note. Victimization was self-reported at T1. Defending T1, Popularity T1, and Social Preference T1 were cluster-mean centered. * *p* < .05. ** *p* < .01, *** *p <* .001

At the classroom level, there were significant small positive associations of bullying descriptive norms with defending descriptive norms (*r* = .22) and defending popularity norms (*r* = .16). There were small to moderate negative associations of bullying popularity norms with defending descriptive norms (*r* = − .12) and defending popularity norms (*r* = − .30). There was a small positive association of defending descriptive norms with defending popularity norms (*r* = .17).

The variance of the random intercepts was small but statistically significant, indicating that victimization varied between classrooms (variance < 0.01, *SE*_T1−T2_ = 0.06–0.07). The residual variance was 0.11 (*SE*_T1−T2_ = 0.31–0.34), indicating also substantial variability at the individual level.

#### Concurrent associations

##### Effects of individual-level predictors

**Fig. 1:**
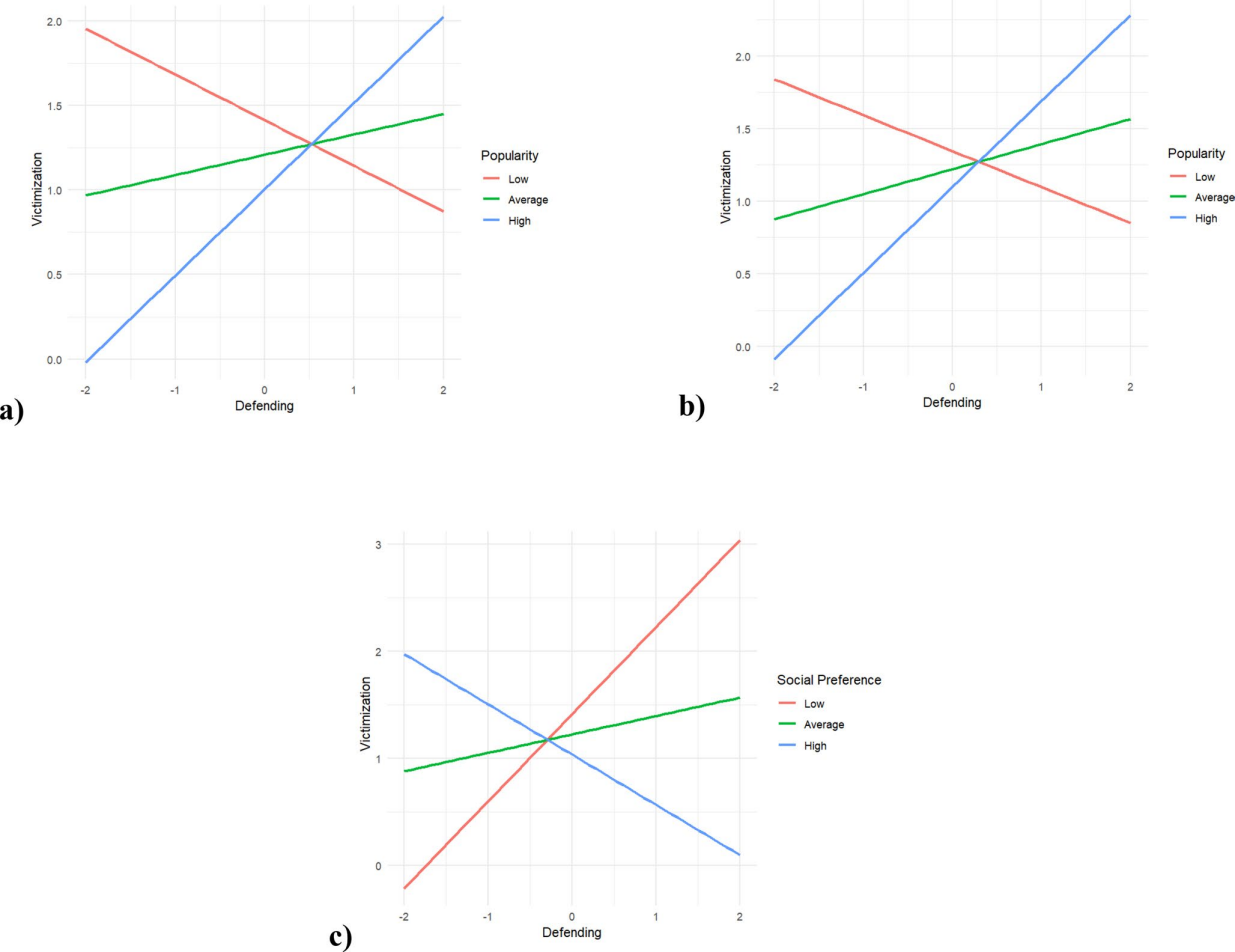
Interactions between **(a)** defending and popularity predicting self-reported victimization T1, **(b)** defending and popularity predicting self-reported victimization T2, and **(c)** defending and social preference predicting self-reported victimization T2. Note. Low and high popularity and preference were defined as M +/- 1 SD

The hypothesis that the association between defending and victimization would depend on children’s popularity and social preference was partly confirmed. Defending was not significantly associated with T1 self-reported victimization as a main effect, β = 0.04, *p* = .814, but the interaction with popularity was significant, β = 0.03, *p* = .006. Simple slope analyses indicated that at low levels of popularity, more defending was associated with less T1 victimization (*b* = -0.56), whereas at high levels of popularity, more defending was associated with more T1 victimization (*b* = 0.22). At average levels of popularity, the association was not statistically significant (see Fig. 1a). In contrast, regarding the role of social preference, the interaction between defending and social preference was not significant, β = 0.00, *p* = .937.

##### Effects of classroom-level predictors

At the classroom level, the expectation that bullying norms (i.e., descriptive and popularity) would be associated with more victimization, whereas defending norms (i.e., descriptive and popularity) would be associated with less victimization, was only partly confirmed. Only bullying descriptive norms were positively associated with T1 self-reported victimization, β = 0.02, *p* < .001. Bullying popularity norms, defending descriptive norms, and defending popularity norms were not significant predictors, and none of the interactions between defending and classroom-level norms were statistically significant βs < 0.02, *ps > .*169.

#### Longitudinal associations

##### Effects of individual-level predictors

The hypothesis that the longitudinal effect of defending on victimization would depend on children’s popularity and social preference was confirmed. While defending at T1 was not significantly associated with T2 self-reported victimization as a main effect, β = 0.05, *p* = .099, the interactions with popularity and social preference were significant.

Regarding the role of popularity, the interaction between defending and popularity was positive and significant, β = 0.03, *p* = .010. Simple slopes showed that at low levels of popularity, more defending was associated with less T2 victimization (*b* = -0.54), whereas at high levels of popularity, more defending was associated with more T2 victimization (*b* = 0.30). At average levels of popularity, the association was not statistically significant (Fig. 1b).

Regarding the role of social preference, the interaction between defending and social preference was negative and significant, β = -0.04, *p* = .001. Simple slope analyses indicated that at high levels of social preference, more defending was associated with less T2 victimization (*b* = -0.22), whereas at average and low levels of social preference, more defending was associated with more victimization (*b* = 0.12 and *b* = 0.52, respectively; see Fig. 1c).

##### Effects of classroom-level predictors

At the classroom level, the expectation that bullying norms (i.e., descriptive and popularity) would predict increases in victimization over time, and defending norms (i.e., descriptive and popularity) would predict decreases in victimization over time was partly confirmed. There was a main effect of bullying descriptive norms at T1 positively predicting self-reported victimization at T2, β = 0.00, *p* = .001. Bullying popularity norms, defending descriptive norms, and defending popularity norms were not significant predictors, and none of the interactions between defending and classroom-level norms were statistically significant, βs = 0.00, *ps >* 0.536.

### Sensitivity Analyses

Several additional analyses were conducted to examine the robustness and generalizability of the findings and to explore whether they depended on specific modeling decisions or informants. First, classroom-level norms indexing the extent to which bullying and defending were associated with social preference were computed (within-class correlations between bullying/defending and social preference; bullying-preference norms and defending-preference norms). Adding these social preference norms to the concurrent multilevel models showed that both norms were associated with self-reported victimization at T1, bullying-preference norms: β = -0.06, *p* < .001; defending-preference norms: β = 0.04, *p* = .030, but did not alter the pattern of effects for defending or its interactions with popularity and social preference (see Table S4). When the social preference norms were added to the longitudinal models predicting victimization at T2, neither norm emerged as a robust predictor: bullying-preference norms: β = -0.01, *p* = .494; defending-preference norms: β = -0.04, *p* = .051, and the main conclusions regarding defending and the status moderators remained unchanged (see Table S4).

Second, to test whether the association between defending and victimization differed between intervention and control schools, an Intervention x Defending interaction term was added to the concurrent and longitudinal models. In both cases, the interaction term was not statistically significant, concurrent model: β = 0.00, *p* = .555; longitudinal model: β = -0.01, *p* = .323, and likelihood ratio tests indicated that including the interaction did not improve model fit, concurrent: χ²(2) = 0.43, *p* = .810; longitudinal: χ²(2) = 2.28, *p* = .320. These results suggest that the structural associations between defending and victimization are similar in intervention and control contexts (see Table S3).

Third, models were re-estimated using peer-reported victimization as the outcome. In the concurrent model predicting T1 peer-reported victimization, defending showed a small but significant positive association with victimization, β = 0.05, *p* = .049, indicating that students who were more frequently nominated as defenders were also perceived by peers as somewhat more victimized (see Table S5). In the longitudinal model predicting T2 peer-reported victimization while controlling for T1 peer victimization, baseline victimization remained a strong predictor, β = 0.63, *p* < .001, but the main effect of defending was not significant, β = -0.02, *p* = .869 (see Table S5). Nevertheless, several interactions between defending and status indicators emerged in these models, consistent with the idea that the reputational consequences of defending are embedded in peer status hierarchies and classroom climates.

Finally, to quantify effect sizes at the model level, marginal and conditional R² values were computed. For the main concurrent model with self-reported victimization, the fixed effects accounted for 14% of the variance, marginal R² = 0.14, and the full model including random intercepts accounted for 17%, conditional R² = 0.17. For the longitudinal model predicting self-reported victimization, the fixed effects explained 30% of the variance, marginal R² = 0.30, and the full model explained 33%, conditional R² = 0.33. For the longitudinal model predicting peer-reported victimization, the fixed effects explained 54% of the variance, marginal R² = 0.54, and the full model explained 59%, conditional R² = 0.59, reflecting the strong stability of peer-reported victimization over time (see Table S2).

In additional models that included self-reported bullying and peer-reported victimization as covariates, the pattern of results was substantively unchanged; for brevity, these models are not shown but are available upon request.

## Discussion

Bullying and defending behaviors unfold within peer ecologies in which individual status and classroom climates jointly shape youths’ experiences of victimization. Prior work has shown that defenders can sometimes incur social costs (Lambe et al., 2019), but has provided limited evidence about the conditions under which defending is protective versus risky, particularly in primary school settings and in relation to different forms of peer status and classroom norms. Addressing this gap, the present study examined concurrent and longitudinal associations between defending and victimization among primary school youth, focusing on popularity, social preference, and classroom bullying and defending norms as potential moderators. Overall, the findings suggest that the social consequences of defending are highly contingent on defenders’ position within the peer group and the broader classroom context.

At the individual level, significant interactions with popularity and social preference indicated that the social consequences of defending varied substantially depending on children’s peer status. Specifically, victimization was particularly high among children low in popularity who engaged in little defending, as well as among highly popular children who engaged in high levels of defending. Thus, whereas defending appeared protective for low-popularity youth, it was associated with increased victimization among high-popularity youth. It might be that engaging in defending may provide low-status youth with opportunities to gain visibility or moral recognition within the peer group, potentially altering how they are perceived, which aligns with prior research suggesting that defending can strengthen affiliative bonds and signal alignment with prosocial group norms (Laninga-Wijnen et al., 2023). However, defending may also disrupt existing dominance hierarchies, particularly if it challenges peers who engage in bullying. When high-status youth intervene, their actions may be perceived as threatening to others’ social standing, potentially eliciting retaliatory responses (Huitsing et al., 2014).

The interaction with social preference revealed a different pattern: Defending was protective for students with high levels of social preference but predicted increased victimization for those low in social preference. Unlike popularity, which reflects visibility and dominance, social preference reflects affiliative ties and peer acceptance (Cillessen & Mayeux, 2004; Rose et al., 2004). Socially preferred defenders may, therefore, benefit from supportive peer networks that buffer against retaliation, whereas students low in social preference may lack such protective alliances. In addition, reciprocal processes of aggression may contribute to this pattern: popular youth who also engage in bullying or other coercive behaviors may become targets of retaliation from those they have previously harmed, whereas well-liked youth tend to be less involved in aggression and are therefore less likely to elicit such repercussions. However, the exact mechanisms through which popularity and social preference provide protection or risks remain unclear and require further study. Importantly, these findings together underscore the importance of distinguishing between different forms of status when evaluating the potential risks and benefits of defending. While socially preferred defenders may benefit from their strong affiliative ties, popular defenders may be more vulnerable to retaliation.

The sensitivity analyses further revealed that defending was positively associated with peer-reported victimization, whereas its association with self-reported victimization was less consistent. This discrepancy may partly reflect that the two measures capture different aspects of victimization: peers reported whether a student had been victimized in the past few months, whereas the self-report scale focused on more prolonged victimization experiences. It is therefore possible that children who defend others are exposed to occasional or short-term victimization that peers notice, but that defenders themselves do not interpret or report as chronic victimization. This pattern highlights the importance of considering differences between informants and measures when examining the social experiences of defending students. In contrast to individual-level interactions, classroom norms did not moderate the association between defending and victimization, either concurrently or over time. Thus, the social risks associated with defending did not depend on whether classrooms were characterized by stronger bullying or defending norms. However, classroom-level bullying descriptive norms did show a consistent main effect: classrooms in which bullying behavior was more prevalent were characterized by higher levels of victimization, both concurrently and over time. This finding suggests that victimization is partly embedded in broader classroom dynamics, such that environments in which bullying is common may structurally sustain elevated levels of risk for students. Importantly, these contextual effects operated independently of defending behavior. Whereas individual status shaped whether defending was associated with protection or increased risk, classroom bullying norms appeared to influence overall levels of victimization rather than the specific social consequences of defending. This distinction underscores that individual- and classroom-level processes may play complementary but distinct roles: peer status shapes interpersonal risks, while descriptive bullying norms reflect broader contextual contexts that affect victimization more generally. Finally, the additional classroom-level norms indexing the association between bullying/defending and social preference showed small and not entirely straightforward associations with victimization; we therefore treat these findings as exploratory and present them primarily as complementary to the main models.

The absence of significant cross-level interactions suggests that the alignment between defending behavior and classroom norms may not be the primary mechanism determining defenders’ vulnerability, at least within the present design. The consequences of defending may be more strongly determined by children’s individual social resources than by broader normative climates. Developmental factors may also help explain the lack of moderating effects. In late childhood, classroom norms around bullying and defending may still be emerging and inconsistently enforced. Such norms may be strong enough to influence overall levels of bullying and victimization - consistent with the main effects of bullying descriptive norms - but not yet sufficiently crystallized to systematically buffer or penalize individual defenders, as has been observed more clearly in adolescent samples. Moreover, the content of high-status norms may be more prosocial in late childhood than in adolescence. In primary school, being good at schoolwork or behaving kindly toward classmates is often admired, whereas in secondary school, similar behaviors may sometimes be dismissed as “nerdy” or uncool. Likewise, bullying and toughness tend to be more strongly valorized as markers of status during adolescence than in childhood. Age differences in both the crystallization and the content of peer norms may therefore help explain why we found only limited evidence that classroom norms moderate the links between defending and victimization in primary school.

Alternatively, classroom norms may operate in more complex ways than captured by the current models, for example, through three-way interactions between defending, individual status, and classroom norms. Because such higher-order interactions were not tested in the present study, firm conclusions about the combined influence of individual- and classroom-level factors cannot yet be drawn and warrant further investigation.

### Theoretical Implications

The present findings contribute to a more detailed understanding of defending and its consequences within peer ecologies by demonstrating that its social consequences depend strongly on children’s position in the group. Rather than exerting uniform effects, defending appears embedded in existing peer hierarchies. At the same time, classroom-level bullying descriptive norms were associated with higher levels of victimization overall, indicating that peer dynamics operate at multiple levels: individual status shapes the risks associated with defending, whereas classroom climates characterized by frequent bullying elevate general levels of victimization.

The distinction between popularity and social preference further refines theoretical models of defending. Popularity and social preference represent different dimensions of status, visibility, and influence versus acceptance and affiliation (Cillessen & Mayeux, 2004), and these dimensions were associated with distinct patterns of risk and protection. Whereas defending was protective for children low in popularity, it predicted increased victimization among highly popular youth. Similarly, social preference shaped the extent to which defending was associated with protection versus risk. These findings highlight that status is multidimensional and that different forms of social status may operate differently in the context of defending. This perspective aligns with frameworks such as Resource Control Theory (Hawley, 1999), which distinguishes between different pathways to social influence and suggests that the social consequences of behavior may depend on the type of resources underlying one’s status. Some popular youth maintain their position by combining prosocial behaviors with more coercive or aggressive strategies. For these adolescents, openly defending victimized peers may be seen as incompatible with a “tough” or intimidating image. Showing kindness - especially toward low-status classmates who are often targets of bullying - can be interpreted by some peers as a sign of weakness or as evidence that the defender is not as formidable as previously thought. Such behavior may therefore invite status challenges from rivals who see an opportunity to contest the defender’s position, or provoke retaliation from those whose bullying is being constrained. In this way, defending may inadvertently increase the social exposure of popular youth and heighten their risk of being targeted, even as it can enhance their moral standing in the broader peer group.

Beyond differences in available social resources, the meaning that peers attribute to defending may also depend on the defender’s status. When defending is enacted by socially preferred youth - who are broadly liked and embedded in dense affiliative networks - it may be interpreted as authentic care for victims and as consistent with their prosocial reputation. Such behavior is likely to strengthen their standing as morally engaged group members and elicit support rather than retaliation. In contrast, when highly popular youth defend, peers may still interpret their behavior through a dominance lens and question whether the defender is genuinely altruistic or strategically managing appearances. For these youth, defending may be scrutinized more critically and more easily perceived as a sign of vulnerability, thereby increasing the likelihood of social sanctions.

Alternatively, popular defenders may be seen as enforcing group norms in ways that threaten other high-status peers, thereby increasing the likelihood of targeted retaliation. These differences in how defending is perceived may partly account for why defending is protective for socially preferred youth but carries heightened social risk for highly popular defenders.

### Practical Implications

From a practical perspective, the present findings underscore the importance of considering children’s social position within the peer group when designing interventions that promote defending behavior. Defending was not uniformly associated with victimization; rather, its consequences differed depending on children’s popularity and social preference. For children low in popularity, defending was associated with lower levels of victimization, both concurrently and over time. This suggests that encouraging defending among lower-status youth may, under certain conditions, have protective effects and may contribute to shifts in how they are positioned within the peer group. Interventions may therefore benefit from recognizing that defending does not increase risk for socially marginalized youth, and their defending should therefore be encouraged as much as possible.

In contrast, for highly popular youth, defending predicted increased victimization over time. This suggests that even socially influential children may incur social costs when intervening in bullying situations, possibly due to shifts in status dynamics or retaliatory responses. Interventions should therefore prepare students, particularly those in visible or influential roles, for the potential complexities of defending, for example, by promoting collective rather than individual defending strategies.

Although classroom-level norms did not moderate the association between defending and victimization in the present study, the consistent main effects of bullying descriptive norms indicate that classrooms in which bullying is more frequent have higher overall levels of victimization. Thus, even when defending norms do not necessarily buffer individual defenders from social risks, the findings underscore the importance of addressing classroom climates more broadly. For example, interventions aimed at reducing bullying may lower overall victimization risk (Salmivalli et al., 2021), even if such norms do not directly alter the social consequences of defending. In addition, creating supportive peer networks and fostering collective responsibility are still important to ensure that defending behaviors are sustained. Future research should further examine how individual status and classroom climates jointly shape bullying-related processes.

### Limitations and Future Directions

While this study provides valuable insights, certain limitations should be noted. First, although peer nominations are commonly used in bullying research and are considered a valid and reliable indicator of victimization, as they reduce shared method variance and social desirability biases associated with self-report (Cillessen & Marks, 2017), they may still introduce bias highlighted by the fact that different conclusions emerged depending on whether peer- or self-reports were used (Košir et al., 2020). Therefore, future studies could additionally include observational methods or teacher-reported data to triangulate findings.

Second, although the present study examined both concurrent and longitudinal associations between defending and victimization, it did not fully capture the dynamic processes through which defending unfolds over time. We did not, for instance, investigate how the immediate consequences of defending at one time point may shape later trajectories of victimization, or how these trajectories depend on individual and classroom-level characteristics. Future research could examine how short-term effects of defending evolve over time and how they are moderated by contextual factors such as classroom norms for defending and the social status of defenders, in order to provide a more comprehensive understanding of the risks and benefits of defending. Moreover, the longitudinal analyses in this study were observational, and the predictive relations over time cannot be taken as evidence of causality. To determine whether the identified risk and protective factors play a causal role in increasing defending and reducing victimization, experimental research is needed, including intervention trials and micro-trials targeting specific putative mechanisms.

Third, no differentiation could be made between different forms of bullying. However, different types of bullying, such as physical, verbal, relational, and cyberbullying, may involve distinct social dynamics, risk factors, and consequences. For instance, relational bullying may be more closely tied to social status and peer group dynamics than physical bullying (Cañas et al., 2022; Espelage et al., 2018). Moreover, by using a single-item measure for victimization, the assessment of internal consistency and content breadth was limited. Nonetheless, global victimization items have been shown to correlate strongly with multi-item victimization scales, and they are commonly used in large-scale school-based research where assessment time is constrained (e.g., Solberg & Olweus, 2003). Importantly, the present measure was not intended to capture specific forms or contexts of bullying, but rather students’ overall exposure to victimization.

Likewise, the measurement of defending in this study involved some limitations. The peer-nomination item (“Some children help others who are being bullied. They can comfort or defend them.”) was designed to capture a broad range of behaviors that support victims and may also include instances in which children confront the bully. As a result, we could not distinguish between different forms of defending, such as victim-oriented versus bully-oriented defending, even though these forms are likely to involve different social dynamics and risks of retaliation (Meter & Card, 2015; Malamut et al., 2025). In addition, the study did not assess the quality or effectiveness of defending. Rejected children with limited social competence may engage in defending attempts that are ineffective or even counterproductive, thereby increasing their susceptibility to subsequent victimization (Pronk et al., 2015). Future research should therefore examine not only the occurrence and type of defending, but also its quality and effectiveness, and investigate whether the protective and risk factors identified in this study operate differently across various forms and outcomes of defending.

Fourth and finally, the longitudinal analyses relied on an autoregressive approach in which victimization at Time 2 was predicted while controlling for victimization at Time 1. Although widely used, such models do not disentangle within-person and between-person processes (e.g., Lucas, 2023). Consequently, the present findings cannot determine whether the observed associations reflect differences between children (e.g., children who generally defend more than others) or changes within children over time (e.g., periods in which a child defends more than usual). This distinction is theoretically important, as recent research suggests that defending may operate differently at the within- and between-person levels (Malamut et al., 2025). Future studies using designs that allow for the separation of stable between-person differences from within-person fluctuations, such as random-intercept cross-lagged panel models, can further clarify the dynamic processes linking defending and victimization.

## Conclusion

Defending youth who are victimized by bullying is often encouraged as a key strategy for promoting safer peer environments, yet the social consequences of defending for defenders themselves are not uniform. This study addressed an important gap by examining when defending predicts increased versus decreased victimization in primary school classrooms, focusing on the joint roles of individual status and classroom norms. Using longitudinal data from a large, diverse sample of youth, this study highlights that the social consequences of defending are not uniform but depend strongly on children’s position within the peer group. Defending was protective for youth who were well-liked or low in popularity, but carried heightened risk for highly popular youth and those low in social preference, underscoring that defending is embedded in existing peer dynamics rather than operating independently of them. At the classroom level, stronger descriptive bullying norms were associated with higher overall victimization, indicating that defenders’ experiences unfold within broader peer ecologies that vary in general risk, whereas classroom norms did not systematically moderate the defending-victimization association, suggesting that individual social resources may play a more proximal role in shaping the specific social consequences of defending. Together, these findings contribute to a more differentiated understanding of how peer status and classroom climates jointly shape the risks and benefits of defending, and highlight that promoting defending as part of anti-bullying efforts requires attention to both peer dynamics and general classroom environments to ensure that defending is encouraged in ways that are socially sustainable and supported. 

## Supplementary Information

Below is the link to the electronic supplementary material.

Supplementary Material 1

## Data Availability

The datasets generated and analyzed during the current study are not publicly available due to institutional regulations but are available from the corresponding author on reasonable request.
