# Supplementary material for: Defending is a Balancing Act: The Consequences of Defending Victims of Bullying Depend on Defender’s Popularity and Social Preference
Source: J Youth Adolesc. 2026 May 30;55(8):1999–2013. doi: 10.1007/s10964-026-02363-4 (PMC13442617; doi:10.1007/s10964-026-02363-4)
Supplement: Supplementary file 1 [file 10964_2026_2363_MOESM1_ESM.docx]

**Supplemental Materials**

**Table S1**

*Baseline Differences Between Retained and Drop-out Students*

| **Variable** | **Group** | **M** | **SD** | **t** | ***p*** | ***d*** |
| --- | --- | --- | --- | --- | --- | --- |
| Age (years) | Drop-out | 11.59 | 1.30 | 47.69*** | <.001 | 1.11 |
|  | Retained | 10.13 | 1.31 |  |  |  |
| Defending | Drop-out | 0.16 | 0.14 | -2.52* | .012 | -0.06 |
|  | Retained | 0.17 | 0.13 |  |  |  |
| Popularity | Drop-out | -0.00 | 0.32 | 4.52*** | <.001 | 0.11 |
|  | Retained | -0.04 | 0.27 |  |  |  |
| Social Preference | Drop-out | 0.19 | 0.25 | -0.55 | .580 | -0.01 |
|  | Retained | 0.19 | 0.24 |  |  |  |
| Victimization | Drop-out | 0.66 | 0.47 | -5.25*** | <.001 | -0.24 |
|  | Retained | 0.77 | 0.42 |  |  |  |
| Boys | Drop-out | 52.6% | - | - | - | - |
|  | Retained | 50.1% | - |  |  |  |

Note. Retained = students with self-reported victimization at both T1 and T2; Drop-out = students without T2 self-reports. *d* = Cohen’s. * *p* <.05. ** *p* < .01, *** *p <* .001.

**Table S2**

*Model-level Effect Sizes (R²) for Main Multilevel Models*

| **Outcome** | **Model** | **Marginal R²** | **Conditional R²** |
| --- | --- | --- | --- |
| Self-reported Victimization at T1 | Concurrent model | .14 | .17 |
| Self-reported Victimization at T2 | Longitudinal model | .30 | .33 |
| Peer-reported Victimization at T2 | Longitudinal model | .54 | .59 |

Note. Marginal R² reflects variance explained by fixed effects; Conditional R² reflects variance explained by both fixed and random effects (Nakagawa & Schielzeth, 2013).

**Table S3**

*Tests of the Intervention x Defending Interaction in Concurrent and Longitudinal Models Predicting Self-Reported Victimization*

| **Model Type** | **Outcome** | **Interaction term** | **β** | ***b*** | **SE** | ***p*** | **Δχ²(2)** | **p(Δχ²)** |
| --- | --- | --- | --- | --- | --- | --- | --- | --- |
| Concurrent | Self-reported Victimization T1 | Intervention * Defending | 0.00 | -0.04 | 0.07 | .555 | 0.43 | .810 |
| Longitudinal | Self-reported Victimization T2 (controlling for T1) | Intervention * Defending | -0.01 | -0.09 | 0.09 | .323 | 2.28 | .320 |

Note. Δχ²(2) values refer to likelihood ratio tests comparing models with and without the Intervention * Defending interaction term (df = 2). All models controlled for age, gender, classroom size, and intervention status; longitudinal models additionally controlled for baseline self-reported victimization.

**Table S4**

*Multilevel Models Predicting Self-Reported Victimization with Additional Social Preference Norms*

|  | **Concurrent** | | |  |  | **Longitudinal** | | | |  |
| --- | --- | --- | --- | --- | --- | --- | --- | --- | --- | --- |
| **Predictor** | **β** | ***b*** | **SE** | **t** | ***p*** | **β** | ***b*** | **SE** | **t** | ***p*** |
| Intercept | 0.00 | 1.33*** | 0.05 | 26.46 | <.001 | 0.01 | 0.80*** | 0.06 | 13.18 | <.001 |
| Victimization T1 | - | - | - | - | - | 0.45 | 0.45*** | 0.01 | 35.22 | <.001 |
| Defending T1 | 0.04 | -0.02 | 0.11 | -0.18 | .854 | 0.05 | 0.21 | 0.13 | 1.59 | .111 |
| Popularity T1 | -0.15 | -0.21*** | 0.02 | -12.86 | <.001 | -0.09 | -0.12*** | 0.02 | -6.60 | <.001 |
| Social Preference T1 | -0.21 | -0.35*** | 0.02 | -16.56 | <.001 | -0.11 | -0.19*** | 0.02 | -7.69 | <.001 |
| Bullying Descriptive Norms | 0.11 | 0.85*** | 0.12 | 7.02 | <.001 | 0.06 | 0.48*** | 0.13 | 3.58 | <.001 |
| Bullying Popularity Norms | 0.03 | 0.03 | 0.02 | 1.72 | .087 | 0.02 | 0.02 | 0.02 | 0.96 | .337 |
| Defending Descriptive Norms | 0.00 | 0.01 | 0.08 | 0.07 | .941 | 0.02 | 0.11 | 0.09 | 1.28 | .202 |
| Defending Popularity Norms | 0.00 | -0.00 | 0.02 | -0.10 | .917 | 0.02 | 0.03 | 0.02 | 1.27 | .204 |
| Bullying Social Preference Norms | -0.06 | -0.08*** | 0.02 | -3.70 | <.001 | -0.01 | -0.02 | 0.02 | -0.68 | .494 |
| Defending Social Preference Norms | 0.04 | 0.05* | 0.02 | 2.18 | .030 | -0.04 | -0.05 | 0.03 | -1.96 | .051 |
| Defending T1 * Popularity T1 | 0.03 | 0.42** | 0.14 | 2.94 | .003 | 0.03 | 0.39** | 0.16 | 2.37 | .018 |
| Defending T1 * Social Preference T1 | 0.00 | -0.09 | 0.18 | -0.52 | .604 | -0.03 | -0.56** | 0.20 | -2.73 | .006 |
| Defending T1 * Bullying Descriptive Norms | 0.00 | 0.03 | 0.71 | 0.05 | .961 | 0.00 | -0.28 | 0.82 | -0.34 | .733 |
| Defending T1 * Bullying Popularity Norms | 0.02 | 0.17 | 0.12 | 1.39 | .165 | 0.00 | 0.07 | 0.13 | 0.55 | .585 |
| Defending T1 * Defending Descriptive Norms | 0.00 | 0.44 | 0.47 | 0.93 | .351 | 0.00 | -0.32 | 0.57 | -0.56 | .576 |
| Defending T1 * Defending Popularity Norms | 0.01 | 0.16 | 0.12 | 1.31 | .192 | 0.00 | 0.08 | 0.14 | 0.56 | .575 |

Note. Victimization at T2 was predicted while controlling for victimization at T1. Defending T1, Popularity T1, and Social Preference T1 were cluster-mean centered. * *p* <.05. ** *p* < .01, *** *p <* .001.

**Table S5**

*Multilevel Models Predicting Peer-Reported Victimization with Standardized and Unstandardized Effects*

|  | **Concurrent** | | |  |  | **Longitudinal** | | | |  |
| --- | --- | --- | --- | --- | --- | --- | --- | --- | --- | --- |
| **Predictor** | **β** | ***b*** | **SE** | **t** | ***p*** | **β** | ***b*** | **SE** | **t** | ***p*** |
| Intercept | -0.02 | 0.01 | 0.01 | 0.92 | .356 | 0.00 | 0.02 | 0.02 | 1.49 | .137 |
| Victimization T1 | - | - | - | - | - | 0.63 | 0.66*** | 0.01 | 53.88 | <.001 |
| Defending T1 | 0.05 | 0.05* | 0.03 | 1.97 | .049 | -0.02 | 0.01 | 0.03 | 0.17 | .869 |
| Popularity T1 | -0.28 | -0.11*** | 0.00 | -28.22 | <.001 | -0.08 | -0.03*** | 0.00 | -7.65 | <.001 |
| Social Preference T1 | -0.29 | -0.14*** | 0.01 | -27.59 | <.001 | -0.09 | -0.05*** | 0.01 | -8.48 | <.001 |
| Bullying Descriptive Norms | 0.25 | 0.55*** | 0.04 | 13.92 | <.001 | 0.06 | 0.14** | 0.04 | 3.34 | .001 |
| Bullying Popularity Norms | -0.03 | -0.01 | 0.01 | -1.82 | .070 | 0.02 | 0.01 | 0.01 | 0.86 | .390 |
| Defending Descriptive Norms | 0.07 | 0.10*** | 0.03 | 3.99 | <.001 | -0.02 | -0.03 | 0.03 | -1.22 | .226 |
| Defending Popularity Norms | 0.01 | 0.00 | 0.01 | 0.64 | .526 | -0.00 | -0.00 | 0.01 | -0.36 | .719 |
| Defending T1 * Popularity T1 | 0.09 | 0.33*** | 0.03 | 9.54 | <.001 | 0.03 | 0.13*** | 0.04 | 3.43 | <.001 |
| Defending T1 * Social Preference T1 | 0.02 | 0.10* | 0.04 | 2.35 | .019 | 0.02 | 0.08* | 0.05 | 1.75 | .080 |
| Defending T1 * Bullying Descriptive Norms | 0.00 | -0.10 | 0.17 | -0.58 | .564 | -0.03 | -0.59** | 0.19 | -3.16 | .002 |
| Defending T1 * Bullying Popularity Norms | -0.02 | -0.06* | 0.03 | -2.29 | .022 | 0.02 | 0.05 | 0.03 | 1.56 | .118 |
| Defending T1 * Defending Descriptive Norms | 0.00 | 0.13 | 0.11 | 1.14 | .255 | 0.01 | 0.14 | 0.13 | 1.12 | .262 |
| Defending T1 * Defending Popularity Norms | -0.00 | -0.01 | 0.03 | 0.50 | .619 | -0.00 | -0.03 | 0.03 | -0.85 | .394 |

Note. Peer-reported victimization at T1 and T2 was based on peer nominations and standardized within classrooms. * *p* <.05. ** *p* < .01, *** *p <* .001.
